# The relationship between foveal anatomy and retinal function in oculocutaneous albinism

**DOI:** 10.1007/s10633-026-10130-w

**Published:** 2026-07-03

**Authors:** Uma Balakrishnan, Niamh Wynne, Wanda Pfeifer, Erica Woertz, Joseph Carroll, Arlene V. Drack

**Affiliations:** 1 Carver College of Medicine, University of Iowa, 200 Hawkins Dr., Iowa City, IA 52246 USA; 2 Department of Ophthalmology and Visual Sciences, University of Iowa, Iowa City, IA USA; 3 Cullen Eye Institute, Baylor College of Medicine, Houston, TX USA; 4 Department of Ophthalmology and Visual Sciences, Medical College of Wisconsin, Milwaukee, WI USA; 5 University of Iowa Institute for Vision Research, University of Iowa, Iowa City, IA USA; 6 Department of Pediatrics, University of Iowa, Iowa City, IA USA

**Keywords:** Oculocutaneous albinism, Electroretinography, Retinal physiology, Foveal hypoplasia, Adaptive optics

## Abstract

**Purpose:**

To examine the relationship between multifocal ERG (mfERG) topography and peak cone density measured with adaptive optics scanning light ophthalmoscopy (AOSLO) in persons with albinism (PWA).

**Methods:**

We obtained best corrected visual acuity (BCVA), mfERG, retinal imaging with AOSLO, and optical coherence tomography (OCT) data in seven PWA and one control. The relationship between central peak cone density, mfERG amplitude topography, and foveal hypoplasia (FH) grade was calculated using Spearman rank correlation. Parafoveal cone densities among a subset of PWA were compared to a previously reported control group.

**Results:**

All PWA had variably below average peak cone density, decreased BCVA, flattened mfERG topography, and FH. Higher peak cone density significantly correlated with higher peripheral mfERG amplitudes (*r*^2^ = 0.73, *p* = 0.0049), but not central (*r*^2^ = 0.25), and did not correlate with steeper (more normal) mfERG topography (*r*^*2*^ = 0.031). Parafoveal cone densities did not greatly differ outside of the central 2° (within Ring 1) between PWA and those of previously reported controls.

**Conclusions:**

The association of increased foveal cone packing with elevated peripheral, but not central mfERG amplitudes, suggests aberrant post-receptoral circuitry in the macula. The presence of excess connections between cone photoreceptors and bipolar cells, similar to the unrefined circuitry seen in fetal retinae, may underly the electrical dysfunction seen in these PWA, and explain why, even with milder FH and denser cone packing, normal visual acuity is often not achieved.

**Supplementary Information:**

The online version contains supplementary material available at https://doi.org/10.1007/s10633-026-10130-w.

## Introduction

Visual impairment is a common feature across all subtypes of albinism, which comprises a group of genetic disorders of pigmentation. Among the known structural and functional abnormalities observed in the retinae of persons with albinism (PWA), the underdeveloped foveal pit has been considered a major contributor to decreased visual acuity, although this relationship is incompletely understood. Foveal hypoplasia (FH) can be seen as an absent foveal light reflex on fundoscopy or loss of some to all normal foveal laminar characteristics (i.e., plexiform layer excavation, foveal pit, outer segment [OS] elongation, and outer nuclear layer [ONL] widening) on optical coherence tomography (OCT). The spectrum of foveal morphology displayed by PWA, classified by the Leicester grading scheme proposed by Thomas et al. and refined by Wilk et al., has overlap with the range of foveal pit depth and volume seen in normal controls [1, 2]. This range appears to have some genotypic correlation: in a multi-center study of FH, while individuals with oculocutaneous albinism (OCA) had variable degrees (grades 1–4) of FH, they found that molecular diagnosis of *GPR143*-associated X-linked ocular albinism (OA) and Hermansky-Pudlak Syndrome was associated with more severe FH overall [3]. Although studies have demonstrated a tendency for lower grade (milder) FH to occur with better best corrected visual acuity (BCVA), foveal morphology (seen on OCT) alone cannot predict BCVA: Marmor et al. demonstrated 4 cases of high-grade FH with relatively good visual acuity, Chong, et al. showed individuals with OA with similar appearing foveae having a wide range of visual acuity, and Wilk et al. reported PWA with “near normal” foveal morphology with relatively poor acuity [1–6].

Poor BCVA in albinism may also arise from pathology of the foveal cone mosaic, which is affected by, but not fully a product of, foveal pit morphology. Wilk et al. proposed an “active–passive” hybrid model of cone packing during foveal development: initially, a sub-normal cone density is actively achieved through signaling and growth factors; then, cones are passively packed together as the fovea develops to achieve the final cone density [2]. These individual cone photoreceptors can be visualized in vivo using adaptive optics scanning laser ophthalmoscopy (AOSLO) to quantify the density of cells in this cone-rich region of the retina. The peak cone density in PWA has been shown to be decreased on average compared to normal controls, although these ranges are overlapping, and directly correlates with severity of FH. [2] Correlation of peak cone density with BCVA in inherited retinal degenerations has been variable and does not display a linear relationship. [2, 7–10] Although this variability may be confounded by the fact that individuals with better acuity are the same ones with quantifiable AOSLO imaging, it has been shown that good visual acuity can be preserved until a critical mass of cones are degenerated due to the redundancy of the visual stimulus. [7, 10]

In addition to the structural retinal abnormalities described above, macular electrical function, measured by multifocal electroretinography (mfERG), which predominately records the electrical response of cone bipolar cells rather than cone photoreceptors themselves, is also abnormal in PWA [11, 12]. Hu et al. reported in 16 PWA that the slope of mfERG amplitudes from the center to the periphery (“topography”) is flatter across the macula in albinism compared to normal controls, although mfERG amplitudes do not correlate with BCVA or degree of foveal hypoplasia [13]. This finding was also reported by Kelly & Weiss and Jethani, et al. [14, 15]. The difference in mfERG amplitudes and latencies described by Hu, et al. was not significantly different between individuals with and without nystagmus. Kelly & Weiss found a similar mfERG topography in PWA under anesthesia, supporting that this feature is not an artifact of unstable fixation [14]. In a similar study with individuals with foveal hypoplasia due to *PAX6* mutations, Dangremond et al. discovered flattened macular electrical topography, but in a pattern distinct from the pattern in PWA, suggesting that mfERG topography may be disease specific [16].

The connection of cones to bipolar cells in the parafovea exhibits a highly specialized and unique one-to-one circuitry, creating a “private-line” midget pathway designed to achieve the maximum spatial resolution [17]. The development of this pathway is a poorly understood aspect of foveal maturation, however cone specialization is known to drive bipolar cell signaling [18, 19]. Albinism is associated with abnormal visual pathways at the optic chiasm; however, it is unknown whether the midget pathway is fully developed in this disorder. There has been limited study into the correlation between mfERG and cone density data, and not specifically in PWA [18]. Here, we examined the relationship between macular electrophysiology and foveal cone packing and explore its implications on the pathophysiology of reduced BCVA in this disorder.

## Methods

### Participants

This prospective study was approved by the University of Iowa Hospitals & Clinics (UIHC) and Medical College of Wisconsin (MCW) Institutional Review Boards (IRB #201,805,777 [UIHC], PR000023898 [MCW]). Fourteen PWA and one control were consented and attempted all testing. Diagnosis of albinism was based on a patient presenting with at least three clinical features of albinism including nystagmus, iris transillumination, fundus hypopigmentation and/or documented foveal hypoplasia on OCT, and/or molecular confirmation of a complete biallelic genotype with disease-causing mutations. Clinical data were obtained via prospective chart review. Genetic testing was performed by either the John and Marcia Carver Nonprofit Genetic Testing Laboratory, University of Iowa (Iowa City, IA); Marshfield Clinic Research Institute (Marshfield, WI); the Casey Eye Institute Molecular Diagnostics Laboratory (Molecular Vision Lab, Hillsboro, OR); the Baylor Genetics Laboratories (Baylor Genetics, Houston, TX) or Fairview Diagnostic Laboratories (Minneapolis, MN). Participants underwent testing with AOSLO and mfERG. One patient was excluded based on comorbid color vision anomaly and another was excluded based on a later molecular diagnosis of Waardenburg syndrome. Five PWA were excluded due to insufficient resolution of cones on AOSLO imaging.

Control data for peak cone density measurements (*n* = 58), parafoveal cone density measurements (*n* = 20), and mfERG (*n* = 19) was obtained from our previous studies of unaffected individuals [13, 20, 21]. 

### Multifocal electroretinography (mfERG)

Monocular VERIS mfERG (VERIS version 6.0, Electro-Diagnostic Imaging Inc., Milpitas, CA, USA) were obtained for each eye following ISCEV 2012 instrument protocols [22]. The mfERG protocol was a 4-min 103-hexagon protocol covering approximately 40° in diameter of central retina. The luminance of each hexagon was modulated according to a binary m-sequence, with each frame lasting 13.3 ms and 75 Hz frame rate. Cross-correlation of the m-sequence with the recorded signal for both eyes was mathematically extracted to produce local responses [23]. The m-sequence was divided into 8 equal segments and recording time for each was 27.31 s. The stimulus pattern was displayed on a black-and-white FIMI Philips 21″ monochrome cathode ray tube monitor (model no: MDO 0709BRM, Italy) allowing for the luminance of the stimulus elements in the light state to be 100 cd/m^2^. Each subject’s vision was optimally corrected with a refractor/camera system for the fixed viewing distance of 40 cm. mfERGs were recorded using a fiber electrode. A DTL Plus Electrode (Diagnosys LLC, Lowell, MA) was used and referenced to the ipsilateral earlobe and grounded to the forehead. Before insertion of the fiber electrode, the cornea was anesthetized with 0.5% proparacaine and the non-tested eye was occluded. The total recording time was approximately 4 min divided into eight segments. All participants were required to maintain fixation during each of the segments. Participants were monitored by the examiner using a video camera to ensure fixation. Additionally, participants were allowed to adopt small head turns desired to minimize nystagmus amplitudes [22]. Artifact removal and reduction filters were used, as well as a low pass filter setting of 100. The waveforms recorded were amplified and automatically band pass filtered. When a stimulus produced a response outside of the filter range, for example, blinks and large eye movements, it was automatically detected by the system as an artifact and rejected and repeated. To calculate mfERG slope between rings we used the following equation:

$$mfERG\;{\text{ }}slope\, = \,\ln {\text{ }}\left( {Amplitude_{{Ring{\text{ }}1}} /{\text{ }}Amplitude_{{Ring{\text{ }}6}} } \right){\text{ }}*{\text{ }}\left( {1/\left( {6{-}1} \right)} \right).$$

### Adaptive optics scanning laser ophthalmoscopy (AOSLO)

Images of the photoreceptor mosaic were acquired using a custom-built, multi-modal AOSLO with a 790-nm super-luminescent diode light source (SLD; Superlum, Carrigtohill, Cork, Ireland). Participants included in this analysis had resolvable cones at the peak in at least one eye; if both eyes were resolvable then the dominant eye was selected for analysis based on the Miles Eye Dominance Test. Prior to imaging, pupil dilation was achieved in the eye of interest with tropicamide (1%) and phenylephrine (2.5%). AOSLO images were registered and montaged as previously reported [2, 20, 24]. The location of peak cone density was determined by mapping cone density over a 300 × 300 µm region of interest (ROI, placed to include the subjective location of peak cone density) using a previously described custom script [25]. Up to 10 smaller ROIs were then extracted from each montage, using the peak cone density locus as the foveal center (0,0) for that respective montage. The ROI size scaled with increasing eccentricity to account for the normal decrease in cone density as follows along the temporal meridian: 0°, 0.5°, and 1° (37 × 37 µm); 1.5°, 2°, and 4° (50 × 50 µm); 6° and 8° (75 × 75 µm); 10° and 15° (100 × 100 µm). Cones in each ROI were semi-automatically identified by a single observer (JC) in a masked fashion (Mosaic, Translational Imaging Innovations), from these coordinates the bound cone density was calculated. Not all locations in all montages had sufficient image quality for unambiguous cone identification. Due to the presence of rod photoreceptors in locations beyond 2 degrees, split detection, rather than confocal, images of cone inner segments were used for easier, more reliable disambiguation of rods and cones [26].

To account for different axial lengths among participants and directly compare to mfERG amplitudes (nV/deg^2^), linear cone densities (cones/mm^2^) were converted to angular density (cones/deg^2^) using the retinal magnification factor (RMF): *angular density* = linear density * (RMF/1000).^*2*^, where *RMF* = axial length * 291/24 [27].

Fixation stability during AOSLO testing was described both by qualitative observation of eye movements (no, minimal, or severe nystagmus) and quantitatively using the bivariate contour ellipse area (BCEA) in the Macular Integrity Assessment (MAIA) microperimeter. Fixation stability rating was defined as previously described by Fujii, et al. based on the percentage of fixation points that were with a 1−(P1) or 2-degree (P2) circle (unstable: P1 and P2 < 75%; relatively unstable: P1 < 75% and P2 > 75%; and stable if P1 and P2 > 75%) [28].

### Optical coherence tomography (OCT)

Data was obtained using the Heidelberg Spectralis OCT (Franklin, MA) or the Zeiss Cirrus OCT (Dublin, CA). Foveal hypoplasia (FH) grade was assessed and reconciled between 3 reviewers (UB, NW, AVD) using the scale developed by Thomas et al. and Wilk et al. [1, 2]. Grade 1 FH is the mildest morphology grade and indicates presence of a normal (1A) or shallow (1B) foveal pit without complete plexiform layer excavation. The following grades indicate loss of the following characteristics in a step-wise fashion: the foveal pit (Grade 2), OS elongation (Grade 3), and ONL widening (Grade 4). Grade 4 FH is synonymous with fovea plana.

### Statistical analyses

BCVA was converted to the logMAR scale via the following calculation: *logMAR* = -log(*Decimal acuity*) + 0.02X–0.02Y; where X = number of letters missed on the line and Y = number of letters correctly identified on the following line. Data analysis was performed in Microsoft Excel for MAC (v16.87, Microsoft; Redmond, WA, USA). Spearman rank correlation was used to assess association of cone packing with BCVA, mfERG amplitudes, and mfERG amplitude rate of change from Ring 1 (R1) to Ring 6 (R6). Nonparametric testing was performed. One-sided p-values are reported because of our hypothesis that there is a directional, positive correlation between higher cone density with higher mfERG amplitude and steeper (higher) mfERG amplitude slope.

## Results

### Cohort overview

Seven PWA and one control had interpretable AOSLO, mfERG, and OCT data and these are summarized in Table 1 along with select clinical features (See Supplemental Figs. 1–2). MfERG and OCT data are listed only from the eye selected for AOSLO analysis. Among PWA, there were 4 males and 3 females with mean age 25.1 years (14–48 years, SD 15.0 years). PWA are classified in Table 1 according to the diagnostic criteria outlined by Woertz et al. with “confirmed” (at least 2 out of 3 clinical features [nystagmus, ocular hypopigmentation, and FH] plus molecular diagnosis), “likely” (1 disease-causing mutation and all 3 clinical features), or “suspected” (all clinical features present without a disease-causing mutation in known albinism genes) albinism [29]. Four out of 7 PWA had “confirmed”, 2 had “likely” and 1 had “suspected” OCA. Six out of 7 PWA had confirmed or likely OCA1, and one PWA (JC_11899) was affected by OCA2. Genetic testing for one PWA (AD_12015) did not reveal any confirmed pathogenic mutations, however this PWA is a part of a multi-generational family with multiple similarly affected individuals. The unaffected control subject AD_11298 was homozygous for c.1205G > A (p.Arg402Gln), a known hypomorphic allele, had normal BCVA (20/16 + 2), foveal pit volume (0.12 mm^3^), mfERG amplitudes (R1 = 96.25 nV/deg^2^), and peak cone density (148,832 cones/mm^2^).

**Table 1:**
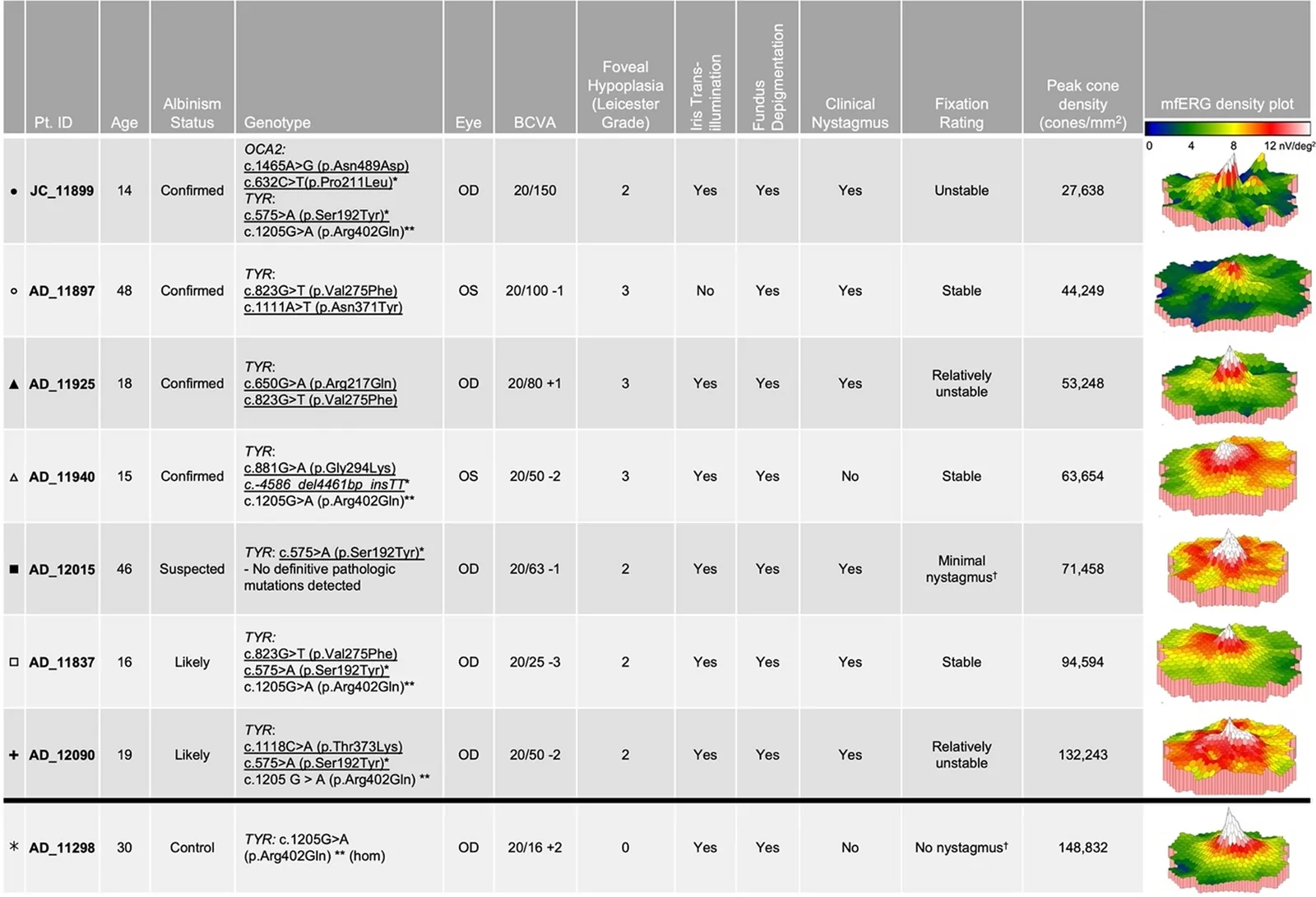
Summary of demographic information, albinism status per Woertz et. al, [29] molecular genetic testing (pathogenic mutations underlined, likely disease-causing underlined with asterisk (*), ** = known hypomorphic allele), Snellen BCVA of the study eye (−*x* or + *x* used to denote the number of letters missed on the line [−] or additional letters seen on the next line [ +]), degree of foveal hypoplasia (see Supplemental Fig. 1), presence of clinical nystagmus, and Macular Integrity Assessment (MAIA) microperimeter rating of fixation stability († = MAIA data not available [see Supplemental Table 1], qualitative assessment of eye movement during AOSLO imaging is given instead), calculated peak cone density, and mfERG density plot with false color legend given (additional mfERG tracings in Supplemental Fig. 2)

Mean logMAR BCVA of the eye tested on AOSLO was 0.53 (Snellen equivalent: 20/68) among PWA. BCVA ranged from relatively normal (20/25–3) to severely decreased (20/150). All PWA had some degree of foveal abnormality (median Leicester grade 3). Although degree of FH and BCVA were generally associated, a notable exception is JC_11899 with relatively low grade FH (2) and BCVA 20/150. PWA demonstrated a wide range of peak cone density (69,583 ± 34,782 cones/mm^2^; range 27,638–132,243 cones/mm^2^), which is significantly lower but slightly overlaps normal controls reported by Cava, et al. (180,286 ± 25,436 cones/mm^2^; range 122,710–247,710 cones/mm^2^, *p* = 5.5 × 10^–14^) [20]. In our limited cohort, higher peak cone density significantly correlated with more normal foveal morphology although the confidence interval includes zero (*r*^*2*^ = 0.72, 95% CI [-0.99, 0.24], *p* = 0.015).

One out of the seven PWA did not have clinical nystagmus (AD_11940). However, individuals with nystagmus did not always display the same amount of fixation instability in free space as during clinical imaging. MAIA was available for 6 of 7 PWA and control AD_11298 (Supplemental Table 1). Two PWA (AD_11897 and AD_11837) demonstrated no nystagmus and stable fixation on MAIA. JC_11899 was the only PWA with severe nystagmus and unstable fixation. There was no eccentric fixation in this cohort and, among those with MAIA, 0% fixation losses. There was no relationship between fixation area (BCEA@95%) and Ring 1 mfERG amplitude, which arises from the central 5 degrees from the fovea, approximately 65 deg^2^ (*r*^*2*^ = 0.056).

**Fig. 1:**
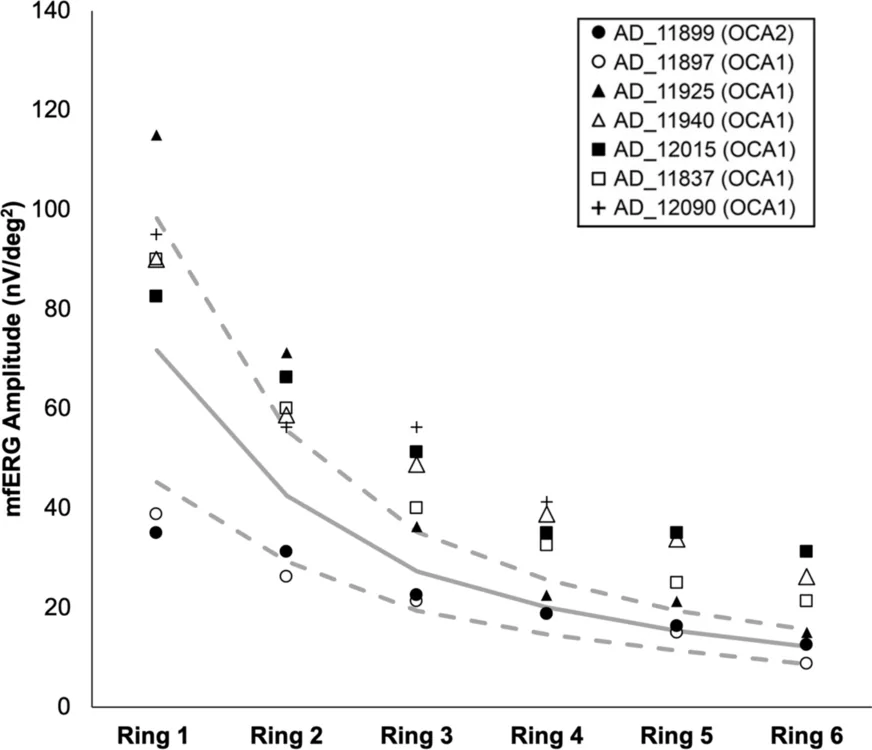
Multifocal electroretinogram amplitudes of 7 persons with albinism (PWA) plotted with normal participants (gray line with ± one standard deviation [dotted lines] from Hu, et al. n = 19 [13])

Average slope from mfERG amplitudes in R1 to R6 (peripheral 25–30º) was −0.266, which is consistent with the findings by Hu, et al., where PWA, with or without nystagmus, have flattened topography (slope = −0.228) compared to normal controls (slope = −0.322) [13]. Figure 1 plots mfERG amplitudes from Ring 1 to Ring 6 in comparison to the controls reported by Hu et. al to visually demonstrate this difference in slope. The flattened topography in our cohort did not have an association with BCVA (*r*^*2*^ = −1.3 × 10^–3^).

### Higher peak cone density is associated with abnormally high peripheral mfERG amplitudes

**Fig. 2:**
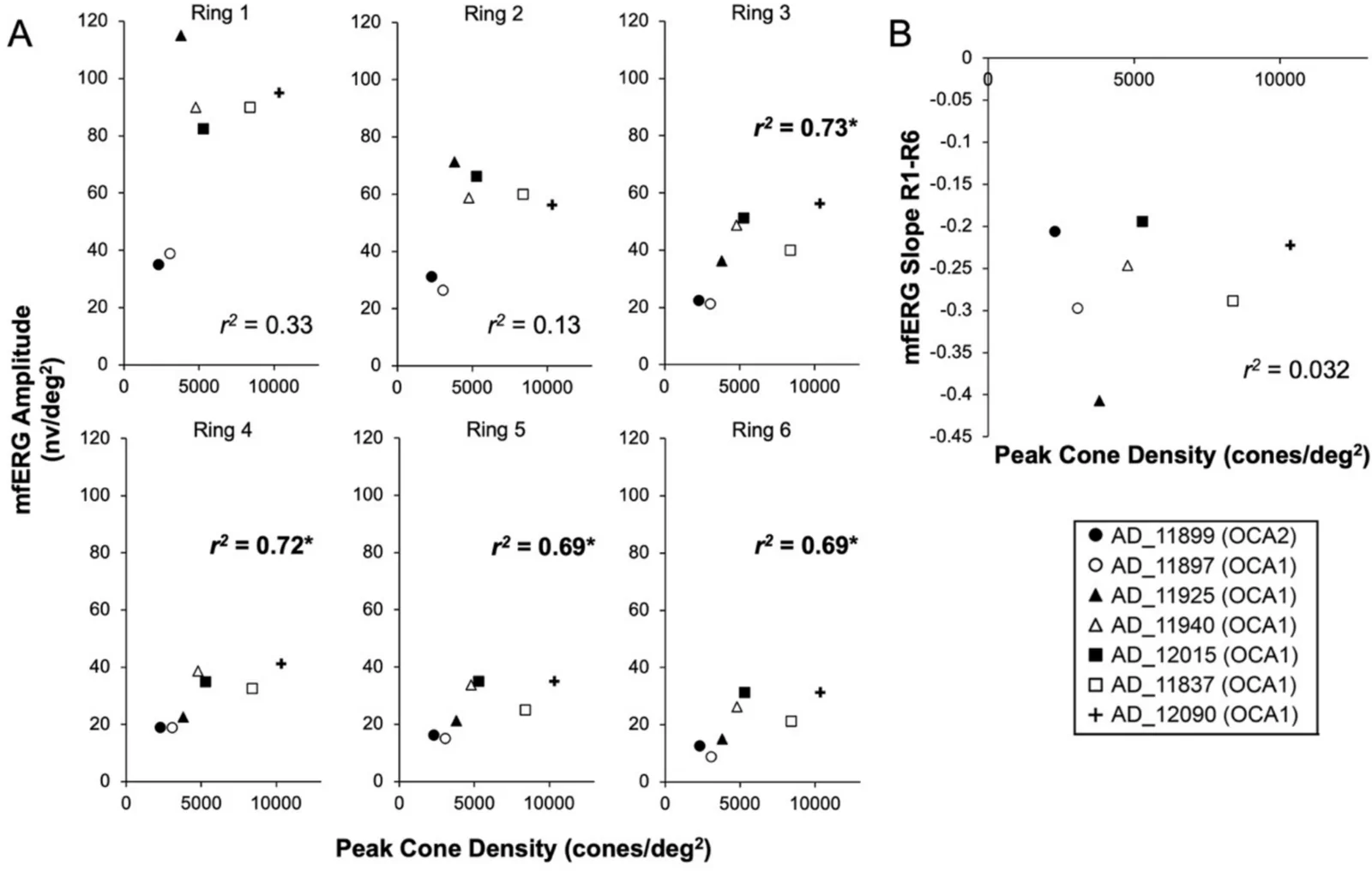
Comparison of mfERG measurements and central cone packing: (**A**) Relationship between mfERG amplitudes and peak cone density in Ring 1 (R1) through Ring 6 (R6). Higher peak cone density is significantly correlated with higher peripheral amplitudes in Rings 3 through R6. (**B**) Rate of amplitude decline from R1 to R6 plotted against peak cone density does not show a significant relationship between cone packing and mfERG topography

Higher central peak angular cone density was not significantly associated with higher amplitudes in the central-most mfERG rings (Ring 1: *r*^*2*^ = 0.33, Ring 2: *r*^*2*^ = 0.13). However, higher central peak cone density was associated with higher mfERG amplitudes in Ring 3 (*r*^*2*^ = 0.73, *p* = 9.8 × 10^–3^), Ring 4 (*r* = 0.72, *p* = 0.012), Ring 5 (*r*^*2*^ = 0.69,* p* = 0.016), and R6 (*r*^*2*^ = 0.69, *p* = 0.016), as well as with higher averaged peripheral amplitudes (Ring 4-Ring 6 average: *r*^*2*^ = 0.73, *p* = 4.9 × 10^–3^) (Fig. 2A). Finally, higher central peak cone density was not associated with a steeper, more physiologic mfERG slope from R1–R6 (*r*^*2*^ = 0.032) (Fig. 2B). (Data summarized in Supplemental Table 2).

### Cone density and mfERG topographies are not analogous

**Fig. 3:**
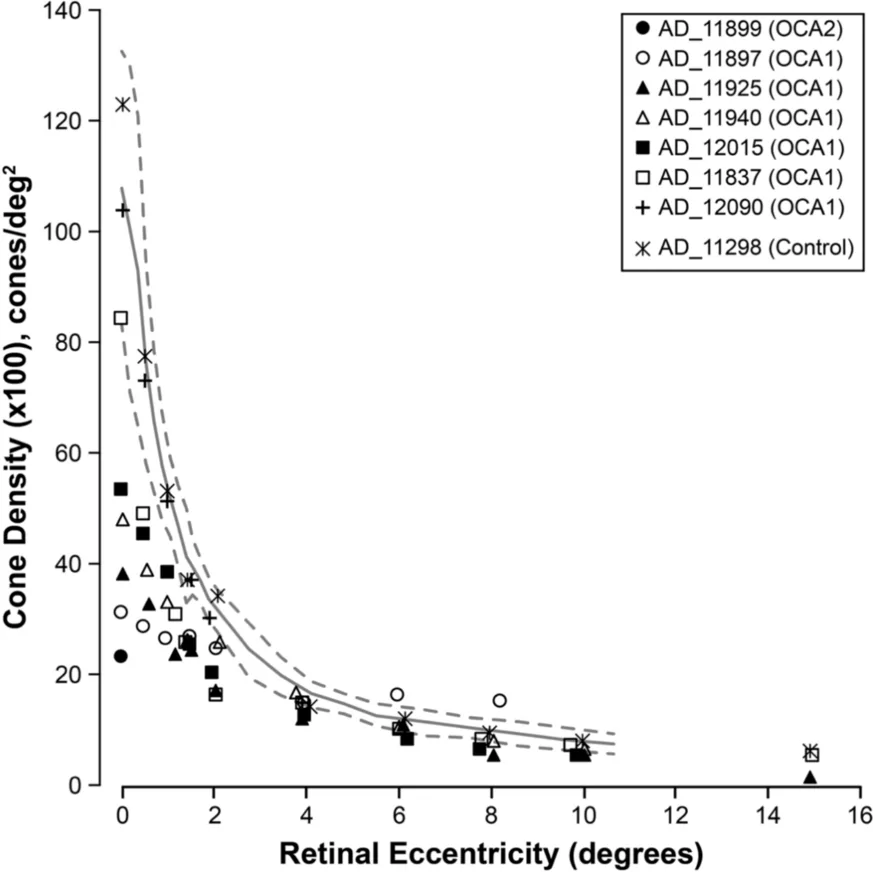
Cone densities of 7 persons with albinism (PWA) plotted over a range of eccentricities compared to control AD_11298 and normal participants (gray line with ± three standard deviations [dotted lines] from Cooper, et al. *n* = 20). [21] PWA have decreased cone densities within the central 2°, measurements beyond that appear similar to the control participants

Parafoveal cone density measurements were able to be calculated for five participants: AD_11940 and AD_11925 to Ring 2 (5-10º), AD_11837 and AD_12015 to Ring 3 (10-15º), and AD_11298 (unaffected control) to Ring 4 (up to 17.1º). Parafoveal cone density measures were limited either by the maximum eccentricity of images obtained or by where cones were able to be resolved. Among PWA, parafoveal cone densities were lower than previously reported in controls within the central 2º, but indistinguishable from controls further from the location of central peak cone density (Fig. 3). [30] Exponential decrease in cone density was compared to the slope in amplitudes from Ring 1 to the most peripheral ring with available cone density measurements, and we did not observe a tendency for steeper rate of decline in cone density to occur with steeper mfERG topography among the four PWA with measurements beyond Ring 1 (> 5º).

## Discussion

In this study, we examined the correlations between foveal anatomy and retinal electrical function in oculocutaneous albinism (OCA). This cohort displayed a wide range of peak cone densities and elevated peripheral mfERG amplitudes, consistent with previous small studies in this population [2, 13]. We observed a complex relationship between foveal cone packing and retinal electrophysiology that may indicate that aberrant connections between macular cone and bipolar cells is part of the origin of the poor visual acuity observed in this family of genetic disorders. Although we expected higher, more normal central peak cone density to correlate with steeper, more normal mfERG topography, instead we found that higher central peak cone density is associated with higher peripheral, and not central, mfERG amplitudes in OCA.

Prior to an analysis of these unexpected findings, it is important to consider the effect of unstable fixation on the data presented. Chu, et al. found that large (4 and 6 degrees), prolonged (2 s), and voluntary disruptions in fixation can lead to decreased central mfERG amplitudes [31]. However, these voluntary eye movements are distinct from nystagmus, and Chu et al. did not report any effect on peripheral mfERG amplitudes where we had our most interesting findings. It is important to note that even healthy individuals with normal visual acuity only maintain fixation 51% of the time [31, 32]. PWA in this study appeared to display less severe nystagmus during testing than in free space: AD_11897 and AD_11837 had clinical nystagmus, but measures of fixation during AOSLO testing were within the normal range [28]. Additionally, we demonstrate that mfERG patterns in this study are consistent with Hu et al., where central mfERG amplitudes overlap with a normal range, peripheral amplitudes appear higher than those of controls, and mfERG slope from R1 to R6 was flattened [13]. This flattened mfERG topography has also been seen in PWA under anesthesia by Kelly and Weiss, free from the effect of nystagmus [13, 14].

Nystagmus does not affect the reproducibility of AOSLO data because PWA have a contiguous cone mosaic, however unstable fixation can lead to unsuccessful image acquisition and unresolvable cones. In one study with 82 patients with diagnosed, likely, or suspected albinism, AOSLO was successful in 57% of patients; older age and better VA were significant predictors of ability to measure cone packing [9]. Our present study excluded participants without analyzable AOSLO images (5/14); therefore, this cohort is subject to selection bias toward PWA with less severe OCA phenotypes and may not be representative of the entire albinism population [9]. Particularly in the parafoveal cone density analysis, we were limited because peripheral cone packing is technically difficult to capture and resolve in PWA. Even in the four PWA where peripheral cone counts were attempted, measurements do not extend into Rings 4–6 where we observed a significant relationship between central cone density and mfERG amplitudes.

If we are to accept the presented data as reliable despite fixation instability, the macular electrical dysfunction described both by Kelly & Weiss and Hu, et al. is not a byproduct of decreased central cone packing [13, 14]. A higher peripheral mfERG response could mean that either there is (1) increased stimulus to the bipolar cells, for example from light scatter effect, (2) increased peripherally located cones, or (3) increased number of bipolar cells receiving input from each cone. Light scatter from optical media opacities is known to decrease central and increase peripheral mfERG amplitudes [33]. Indeed, the supranormal full-field ERG response seen in PWA is often attributed to light scatter due to fundus hypopigmentation [34]. However, degree of hypopigmentation (both iris transillumination defects and fundus pigment) is not reliably correlated with ERG amplitudes: Hu et al. demonstrated two PWA with OCA1, one with “massive” iris transillumination defects and the other with significantly fewer, that had very similar ERG amplitudes compared to each other and to age-matched controls [13]. Future and larger studies with more detailed quantification of hypopigmentation and specifically mfERGs are of particular interest. However, the mfERG pattern seen in this study and in Hu et al. is one where central mfERG amplitudes are not decreased compared to controls, which would be expected from Chan, et al., and it is therefore of interest to explore a physiologic, rather than artifactual, etiology for these findings.

Wolsley et al. found a strong correlation (*r*^*2*^ = 0.96) between co-localized cone density, calculated from a modified Heidelberg retina tomograph, and mfERG amplitudes in the central retinae of two control patients, which was not seen in this current study. [18] In this study of oculocutaneous albinism, we instead demonstrated that PWA do not appear to have increased cone density outside of the central 2° of the fovea and found an absence of a relationship between peak cone density and central mfERG amplitudes, finding instead that central foveal cones, despite their sub-normal density on average in PWA, are co-localized with normal-range R1 mfERG amplitudes. Therefore, we hypothesize that abnormal parafoveal mfERG topography in PWA may be explained by a stronger signal from bipolar cells due to that a supranormal number of synapses to parafoveal cone photoreceptors.

Prior studies have hypothesized that premature and albinotic retinae have aberrant connections between cones and bipolar cells [35, 36]. Zhang et al. demonstrated on histology of a retina at fetal week 14 that cone photoreceptors synapse onto multiple midget bipolar cells, and vice versa, before undergoing synaptic convergence and achieving the one-to-one specialized pathways seen in adults in the parafovea [35]. Woertz, et al. applied this hypothesis to their study comparing adaptive optics and functional MRI data to assess retinotopy among PWA and controls. They demonstrated that cortical magnification underestimated by a model based on foveal cone density. Rather, a model using retinal ganglion cell density was more predictive of cortical magnification, suggesting that foveal cones, despite having decreased density in PWA, may have aberrant connections with downstream signaling cells, including bipolar and ganglion cells [36].

Reports of participants with a history of premature birth have shown overall decreased mfERG amplitudes, however, theoretically a supranormal number of convergent cone-bipolar connections would generate an increased mfERG amplitude [37, 38]. An immature macular circuitry would contain both increased convergent and non-convergent connections, although non-convergent synapses of a single cone photoreceptor with a fixed stimulus to multiple bipolar cells would not be expected to increase mfERG amplitude. The parafovea (> 2mm from the fovea) where private-line connectivity is expected correspond to some of mfERG Ring 2 and all of Rings 3–6. In these peripheral Rings, PWA display elevated mfERG amplitudes despite having apparently normal range parafoveal cone densities. These findings together support a hypothesis that foveal cones in albinism have aberrant connectivity with bipolar cells and that this is the cause of the macular dysfunction described in this disorder.

Higher peak cone density (although still below the normal range) is associated with a more mature fovea, and its correlation of with elevated peripheral mfERG amplitudes in this limited cohort, which we hypothesize is due to immaturity of the midget pathway, is unclear. While midget cell circuitry undergoes its major synaptic remodeling between fetal weeks 14 and 21, foveal cone packing continues post-natally and may be affected by multiple factors, including, but not limited to, the midget pathway itself [35]. We hypothesize connectivity is anomalous in albinism, therefore a higher central peak cone density results in more anomalous connections, which are manifest as higher peripheral mfERG amplitudes. The underlying developmental mechanism of this correlation remains not fully explained.

This study has some important limitations in addition to the major consideration of the effect of unstable fixation on the data presented. These include a small sample size and limited genetic diversity. Because albinism represents a group of disorders of pigmentation with significant phenotypic diversity, the findings of individual small cohorts with predominately oculocutaneous albinism may not be generalizable to all syndromes and subtypes with this condition. Wilk, et al. assessed the genotype–phenotype correlation between molecular genetic diagnosis and cone packing: their study contained one subject with X-linked ocular albinism who had the lowest peak cone density [2]. This type of correlation is limited in our study and others because of the genotypic ambiguity (reportedly up to 25%) that is common among PWA [39]. Future studies extending this analysis of correlation between mfERG amplitudes and foveal cone density in larger cohorts of PWA will be of interest to demonstrate whether these relationships are consistent across different syndromes and subtypes of albinism.

Overall, this study contributes to existing literature describing albinism as a condition of variably arrested foveal development [40]. PWA exhibit multiple features of an immature central retina that have been seen in premature infants, i.e. foveal hypoplasia and decreased peak cone density, and we now propose that the post-synaptic macular circuitry may also be immature, leading to electrical dysfunction of downstream cells assayed by mfERG. Although within this cohort BCVA was only significantly correlated with peak cone density, we agree with prior studies that cone density alone is not highly predictive of BCVA, as seen by the outlier AD_11837 who had severely reduced peak cone density (94,594 cones/mm^2^) and almost normal BCVA (20/25). The combination of retinal and posterior visual system abnormalities likely contributes to poor BCVA in albinism, however based on our findings we would suggest further study into the macular cone and bipolar structure and function, with special attention to the areas 10–30^o^ from the foveal center.

## Supplementary Information

Below is the link to the electronic supplementary material.

Supplementary file1 (DOCX 1421 kb)

## Data Availability

No datasets were generated or analysed during the current study.

## Declarations

### Statement on the welfare of animals

No animals were used in this study; it is a human study, performed with IRB approval for the human subjects.
