# Supplementary material for: The relationship between foveal anatomy and retinal function in oculocutaneous albinism
Source: Doc Ophthalmol. 2026 Jul 3;153(2):223–34. doi: 10.1007/s10633-026-10130-w (PMC13619688; doi:10.1007/s10633-026-10130-w)
Supplement: Supplementary file 1 [file 10633_2026_10130_MOESM1_ESM.docx]

***Supplemental Table 1***

Macular Integrity Assessment (MAIA) microperimeter fixation indices were available for 6 out of 8 subjects. BCEA = bivariate contour ellipse area, P1 = percent of fixation points within a 1-degree circle, P2 = percent of fixation points within a 2-degree circle (Rating is based on definitions provided by Fujii, et al. [28])

***Supplemental Table 2***

Spearman rank correlation analysis of relationship between peak cone density with listed variables (one-sided p values are reported and significant if *p* < 0.05)

***Supplemental Figure 1***

Optical coherence tomography images demonstrating foveal structure (refer to Table 1 for FH grading)

***Supplemental Figure 2***

Multifocal electroretinogram for PWA and control AD_11298: single traces (left) and ring averages (right; black = PWA, blue = study age-matched controls, when available)
